# Impact of Ospemifene on Quality of Life and Sexual Function in Young Survivors of Cervical Cancer: A Prospective Study

**DOI:** 10.1155/2017/7513610

**Published:** 2017-07-11

**Authors:** Nicoletta De Rosa, Giada Lavitola, Pierluigi Giampaolino, Ilaria Morra, Carmine Nappi, Giuseppe Bifulco

**Affiliations:** ^1^Department of Obstetrics, Gynecology, and Urology, University of Naples “Federico II”, Naples, Italy; ^2^Department of Public Health, University of Naples “Federico II”, Naples, Italy

## Abstract

**Background:**

Cervical cancer (CC) treatments impact quality of life (QoL) and sexual function (SF) of survivors. Treatment options to reduce sexual dysfunction are limited. The aim of this study was to assess the effectiveness of ospemifene in CC survivors with clinical signs and symptoms of vulvovaginal atrophy (VVA) focusing on their QoL and SF.

**Materials and Methods:**

Fifty-two patients with previous diagnosis of stage I-IIa CC suffering from VVA and treated with ospemifene were enrolled into a single arm prospective study. Patient underwent 6 months of therapy. At baseline and after 6 months all subjects performed Vaginal Health Index (VHI). The SF and QoL were measured by The European Organization for Research and Treatment of Cancer (EORTC) Quality of Life Questionnaire (QLQ) and the Cervical Cancer Module (CXC-24).

**Results:**

After treatment a significant improvement of each parameter of VHI has been demonstrated. Global health status and emotional and social functioning scores improved significantly. On the contrary, general symptoms scales did not show significant difference from baseline data. Sexual activity, sexual vaginal functioning, body image, and sexual enjoyment scores increased significantly.

**Conclusion:**

Ospemifene seems to be effective in decreasing the VVA symptoms in CC survivors.

## 1. Introduction

Gynecologic cancer diagnosis and related surgical or medical treatments deeply impact quality of life (QoL) and sexual function (SF) of patients. QoL and SF are particularly impaired by cervical cancer, which arises in young-adult patients and impacts on sexual beings, body images, and childbearing potential with the consequent induction of severe emotional distress, anxiety, and behavioral disruptions.

Cervical cancer (CC) is the fourth most common cancer in women and the seventh overall with an estimated 528,000 new cases in 2012 [1]. It is diagnosed among relatively young women; 45% of women diagnosed in 2013 were younger than 45 years [2].

CC survivors have more pronounced menopausal symptoms, body image problems, and sexual problems than the general population [3]. A gradual improvement of emotional distress and QoL issues during the first 2 years after diagnosis of CC has been reported with the exception of lymphedema and menopausal symptoms [4]. Around 80% of CC survivors suffer from sexual dysfunction [5], such as the decrease in vaginal sensitivity, reduction of sexual desire, orgasm, and excitation due to vaginal dryness, sore and blood loss, dyspareunia, and vaginal atrophy following treatment [6].

Treatment modalities to reduce menopausal symptoms and sexual dysfunction are extremely limited for gynecologic cancer survivors. The use of hormones (e.g., local or systemic estrogen) poses a potential risk in patients with cancer. Therefore, the preferred first-line therapy for vaginal dryness and dyspareunia is usually nonhormonal treatments, such as moisturizers and lubricants [7]. Unfortunately, they have only partial effects on sexual dysfunction symptoms [8].

However, therapeutic options have now been increased; after over 20 years in development, ospemifene was approved in early 2013 by the US Food and Drug Administration (FDA) for the treatment of moderate-to-severe dyspareunia associated with vulvar and vaginal atrophy (VVA) due to menopause [9].

Ospemifene is an estrogen receptor agonist/antagonist, also known as a selective estrogen receptor modulator (SERM), from the same chemical class (triphenylethylenes) as tamoxifen and toremifene, both of which are used in the treatment of breast cancer. Ospemifene is, in fact, one of the major metabolites of toremifene. It has an agonist effect on the vaginal epithelium and it has an endometrial and breast safety profile, which makes it unique [10, 11].

Ospemifene improved VVA clinical signs substantially both in hysterectomized women [12] and in women with intact uterus [13]. Vaginal visual examination demonstrated actual improvements in vaginal dryness, redness, petechiae, pallor, and mucosal friability with the majority of patients having no or mild VVA clinical signs at week 52 [12, 13].

Ospemifene (60 mg) in the patient cohort referring dyspareunia increased the percentage of patients who experienced improvement (80% versus 64% with placebo; *p* = 0.001), substantial improvement (53% versus 39% with placebo; *p* < 0.001), or relief (63% versus 42.5% with placebo; *p* < 0.001) [14].

The aim of the current study was to assess the effectiveness of the ospemifene in cervical cancer survivors with clinical signs and symptoms of vulvovaginal atrophy (VVA) focusing on their quality of life and sexual function.

## 2. Material and Methods

From January 2016 until July 2016, 56 eligible patients, referred to the follow-up program at the Unit of Gynecology Oncology of University Federico II of Naples, were enrolled into a single arm prospective study. All patients gave their written consent to be enrolled.

To be eligible for enrolment, patients had to meet all of the following criteria:

- Age between 18 and 60 years.
- Previous diagnosis of stage I-IIa cervical cancer.
- Five-year interval from cancer treatment.
- Stable clinical conditions.
- Active sexual life (≥4 vaginal intercourses in the last month).
- Diagnosis of vulvovaginal atrophy (VVA).
- Good comprehension of the administered written questionnaires.

Brachytherapy or radiotherapy were considered as exclusion criteria.

Medical data (including diagnosis, stage of cancer, type of treatment, time since the end of treatment, and physical and/or psychiatric comorbidities) were extracted from medical records.

All cancer diagnoses performed before 2009 were converted according to the last revised FIGO staging of cervical cancer [15].

Before enrolment, all subjects underwent a gynecological examination, Pap smear, evaluation of Vaginal Health Index (VHI), and complete hematochemical tests.

Patients were treated with 60 mg tablet of ospemifene that is taken by mouth once a day for six months. After 6 months patients repeat gynecological examination and evaluation of VHI.

The VHI includes scoring of vaginal moisture, fluid volume, elasticity, pH, and epithelial integrity on a scale of 1 (poorest) to 5 (best) according to the methods of Robert Wood Johnson Medical School [16]. Lower is the score, greater is the atrophy [17].

Before treatment and after 6 months patients were interviewed on QoL and on SF.

The QoL and SF were measured by The European Organization for Research and Treatment of Cancer (EORTC) Quality of Life Questionnaire C30 (QLQ-C30), a 30-item cancer specific questionnaire for assessing the general QoL of cancer patients [18]. The EORTC-QLQC-30 incorporates five functioning domains: physical (PF2), role (RF2), cognitive (CF), emotional (EF), and social (SF); three symptom scales: fatigue (FA), pain (PA), and nausea/vomiting (NV); several single items which assess additional symptoms commonly reported by cancer patients: dyspnoea (DY), insomnia (SL), appetite loss (AP), constipation (CO), and diarrhoea (DI); the perceived financial impact of the disease and treatment (FI); and finally an overall QoL scale (QL2). Validated module specific to tumour site (cervix, CXC-24) was administered in addition to the core questionnaire [19]. This module includes 24 cancer specific items on symptom experiences after cancer treatment including bladder symptoms, vaginal discomfort, abdominal pain, lymphedema, menopausal symptoms, and peripheral neuropathy and on body image and sexual function evaluating sexual activity, sexual enjoyment, sexual and vaginal functioning, and sexual worry.

### 2.1. Statistical Analysis

Data analyses were performed using the SPSS 20.0 software package (SPSS Inc., Chicago, IL, USA). The levels of significance for all tests were set at *p* < 0.01. Data were evaluated for distribution by Shapiro Wilks' test. Since data did not represent a normal distribution, comparison of variables was performed by using Wilcoxon test.

## 3. Results

Fifty-two patients completed the follow-up and were included into statistical analysis.

Clinical data and disease characteristics of the study group were shown in Table 1.

VHI resulted, at baseline, to be poor with a median total score of 10.00. After treatment an improvement of each parameter has been demonstrated (Table 2). In particular, elasticity, fluid volume, epithelial integrity, moisture, and pH showed a significant increase at 6-month follow-up. The overall median total score at 6 months reached value of 16.00 (Table 2).

**Figure 1:**
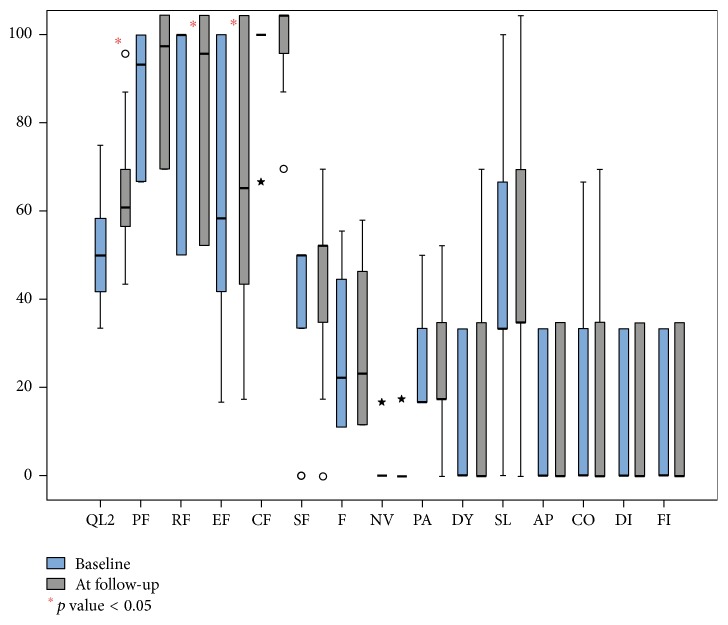
Median values of the EORTC QLQ-C30 subscale at baseline and at 6-month follow-up. QoL: global health status, PF: physical functioning, RF: role functioning, EF: emotional functioning, CF: cognitive functioning, SF: social functioning, F: fatigue, NV: nausea and vomiting, PA: pain, DY: dyspnoea, SL: insomnia, AP: appetite loss, CO: constipation; DI: diarrhoea, and FI: financial difficulties. ○ are extreme values; that is, they do not fall into internal fences. ★ are extreme abnormal values and represent cases/rows with values that exceed three times the height of the boxes.

QoL partially changed at 6-month follow-up. In particular, global health status and emotional and social functioning scores improved significantly (Figure 1). On the contrary, symptoms scales did not show significant difference from baseline data (Table 3).

**Figure 2:**
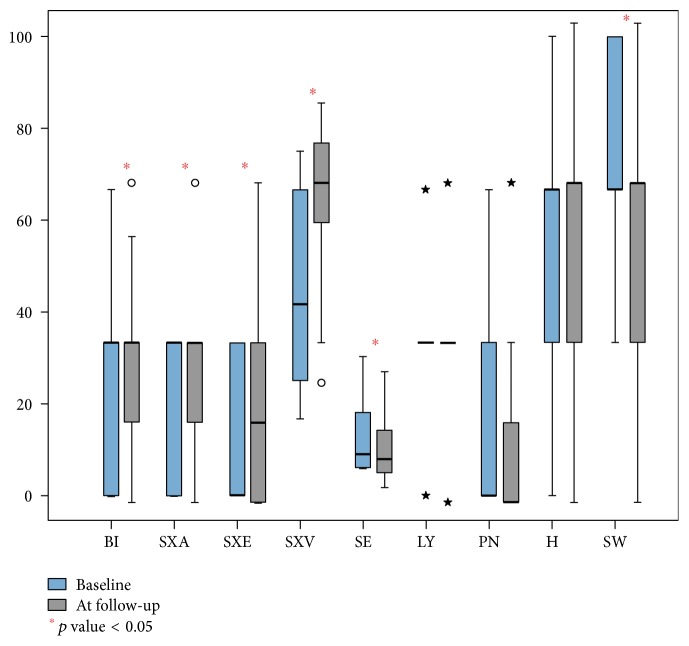
Median values specific cancer module CXC-24 at baseline and at 6-month follow-up. BI: body image, SXA: sexual activity, SXE: sexual enjoyment, SXV: sexual vaginal functioning, SE: symptom experience, LY: lymphoedema, PN: peripheral neuropathy, H: menopausal symptoms, and SW: sexual worry. ○ are extreme values; that is, they do not fall into internal fences. ★ are extreme abnormal values and represent cases/rows with values that exceed three times the height of the boxes.

Functional scale of CXC24 questionnaire, related to sexual function, showed an overall significant improvement. Indeed, sexual activity and sexual vaginal functioning scores increased significantly; body image and sexual enjoyment showed a slight but significant improvement (Figure 2). Regarding symptoms scales, lymphoedema, neuropathy, and menopausal symptoms remained unchanged after treatment but we demonstrated a significant reduction in symptoms experience and sexual worry scores (Table 3).

Patients referred no adverse events during study protocol.

## 4. Discussion

On behalf of our experience, this is the first prospective study that evaluates the effect on VHI, QoL, and SF of ospemifene in cervical cancer survivors.

Our data show an overall significant positive effect of therapy on sexual function of patients affected by vulvovaginal atrophy after cervical cancer treatment. Also QoL partially increases according to the positive effect demonstrated on symptoms experience and on social and emotional functioning.

Sexual function of CC survivors is inferior when compared with general population. About 80% of sexual active CC survivors have sexual dysfunction with negative effect on couple relations and QoL [20–22]. The majority of them show primarily dyspareunia and reduction of sexual desire one-year after surgical treatment [23]. Surprisingly, Lee et al. report, on the contrary, that, compared with healthy women, sexuality was not impaired in cervical cancer survivors who showed no evidence of disease after primary treatment and engaging in sexual activity [24]. However, the study group in this report includes also patients who performed conization, simple hysterectomy, or no surgery and moreover, 79% of the patients have the preservation of ovaries in situ.

Our data show that, about 6 years after treatment, CC patients have important symptoms of VVA. The median total VHI score of our study group is 10.

VVA commonly affects postmenopausal women [22]. It is estimated that up to 40% of postmenopausal women experience symptoms of VVA [23]. These women have a marked impact on sexual functioning, everyday activities, and body image perception [22, 24].

The decline in levels of circulating estrogen associated with the natural aging process or with the oophorectomy, for cancer patients, causes a breakdown of the collagen and elastin fibers in the vagina. The result is an overall loss of vaginal elasticity; the vagina loses its rugae and becomes short and narrow. The epithelium becomes thin and pale. This phenomenon is more pronounced after cancer surgical treatment; indeed, a narrow vaginal opening and a short vaginal stump can worsen VVA symptoms. Moreover, psychological implications of a gynecological cancer diagnosis deeply impact on body image perception and on emotional and social relationship.

We previously report that young gynecological cancer survivors are less sexually active than midlife adults; they suffer much more from their body images, have worse sexual vaginal functioning, and show more severe menopausal symptoms, probably in relationship with rapid body changes following surgical menopause [25].

Our study group of relatively young CC patients (mean age 45 years) shows low baseline scores in global health status and in sexual function; the patients express high level of sexual worry and poor body image. The need of specific intervention in these patients is unquestioned.

The principles of treatment of VVA are the restoration of urogenital physiology and the alleviation of symptoms [26]. Since approval of ospemifene, the therapeutic options for VVA in cancer patients can include only nonhormonal local therapies such as lubricants and moisturizers. However, the 2 principles of treatment usually are not achieved. Lubricants offer a temporary relief of vaginal symptoms, without restoration of urogenital physiology [27, 28]. Moisturizers improve lubrication but have no effect on the overall vaginal maturation index/value (VMI) [29, 30].

Ospemifene is the new nonhormonal proposal for the management of the VVA [10, 11].

In the study group the VHI increases significantly, reaching median value of 16, indicating the positive effect of the ospemifene on vaginal health of CC survivors. This effect was in accordance with the findings of previous studies in general postmenopausal population [12, 31–33]. Indeed, vaginal visual examination demonstrated improvements in vaginal dryness, redness, petechiae, pallor, and mucosal friability at 3 months [31] and 6 months [31, 32] after ospemifene therapy. In women reporting vaginal dryness and dyspareunia, 60 mg of ospemifene reduced both symptoms' severity as compared to placebo [33].

There is no adverse event or tumour recurrence in this study.

Ospemifene appears to have antiestrogenic effects on breast in experimental models. Indeed, in vitro studies showed that ospemifene induced a moderate, dose-dependent growth inhibition of estrogen-dependent MCF-7 cells [34]. In a ductal carcinoma in situ mouse model, cell proliferation was reduced significantly with use of ospemifene [35].

Effects of SERMs on cervical cancer cell are nowadays object of studies. Tamoxifen use exhibited only a marginal protection effect on cervical neoplasia. This finding might be explained by the fact that it acts as an ER agonist rather than antagonist in the uterus [36]. The long-term use of tamoxifen in breast cancer patients actually increases the risks of endometrial cancers [37]. On the contrary raloxifene, which has ER agonistic effect in bone, antagonistic effect in breast, and neutral effect in endometrium [38, 39], similarly to ospemifene, has shown a potential effect in curing both cancer and dysplasia in the cervix in transgenic mouse model [40].

The improvement of VHI in our study group was associated with the improvement in QoL and in SF of the patients. Sexual activity, sexual enjoyment, and sexual vaginal functioning improve significantly at follow-up visit. Sexual worry decreases significantly showing a better predisposition to sexuality.

Menopausal symptoms do not seem to change significantly (in the CXC-24 questionnaire MS correspond to hot flushes); on the contrary symptoms experience that includes vaginal and urinary symptoms shows a significant decrease. Obviously, perception of other cancer specific symptoms does not change significantly after treatment.

A limitation of the current study is the lack of a control group, as the objective was to evaluate changes before and after the ospemifene therapy in CC survivors with VVA as a whole and not to compare its efficacy with other treatment modalities. Thus, a hypothesis of placebo effect cannot be overruled. Moreover, the study does not take into account additional factors that may influence QoL and sexual function, such as marital and economical status of the patients.

Despite the above potential limitations, this study has a considerable strength. This is a prospective study, with a well-defined group of participants: young patients, with previous history of cervical cancer treated by surgery and/or chemotherapy and VVA symptoms.

We excluded patient treated by brachytherapy or radiotherapy to avoid inconclusive results, but for this reason the effect of ospemifene cannot be generalized to all CC survivors.

## 5. Conclusion

Ospemifene seems to be effective in decreasing the VVA symptoms in CC survivors improving VHI, sexual function, and QoL perception of the women. The occurrence of sexual dysfunction has to be recognized by clinicians, which should find the best option treatment for this kind of patient; the ospemifene nowadays is an effective and safety alternative option for the management of VVA in cervical cancer survivors.

Other prospective studies will establish the efficacy of this therapy on other gynecological cancer types and on patients who underwent brachytherapy or radiotherapy.

## Figures and Tables

**Table 1:** Clinical data and disease characteristics of the study group.

Age (years)	45.56 ± 5.44
Stage	
I (a, b)	19 (36.5)
IIa	33 (63.5)
Time since diagnosis (months)	74.63 ± 11.32
Treatment modality	
RH + BSO + PLND	48 (92.3)
RH + BSO + PLND + PALNS	4 (7.7)
Physical comorbidity	
No	39 (75.0)
Yes	13 (25.0)

RH: radical hysterectomy; BSO: bilateral salpingooophorectomy; PLND: pelvic lymph node dissection; PALNS: para-aortic lymph node sampling. Data are shown as mean ± SD or as number (percentage).

**Table 2:** Vaginal Health Index of study group at baseline and after 6 months.

	Baseline	6-month follow-up	*p* value
Elasticity	2.00 [1.52–1.87]	3.00 [3.11–3.59]	<0.001
Fluid volume	2.00 [1.44–1.72]	3.00 [2.48–3.06]	<0.001
pH	2.00 [1.95–2.35]	3.00 [2.66–3.15]	<0.001
Epithelial integrity	2.00 [2.10–2.44]	4.00 [3.48–3.94]	<0.001
Moisture	2.00 [1.99–2.39]	3.00 [2.74–3.22]	<0.001

Total	10.00 [9.54–10.23]	16.00 [15.01–16.41]	<0.001

Data are shown as median [95%, CI].

**Table 3:** Median values of the EORTC QLQ-C30 subscale and specific module CXC-24 at baseline and at 6-month follow-up.

	Baseline	6-month follow-up	*p* value
Global health status/QoL (QL2)	50.00 [50.14–55.31]	58.33 [58.88–65.47]	0.01
Functional scales			
Physical functioning (PF2)	93.33 [82.34–90.74]	93.33 [82.34–90.74]	1.0
Role functioning (RF2)	100.00 [71.67–84.74]	91.67 [72.54–85.15]	0.7
Emotional functioning (EF)	58.33 [59.96–96.78]	62.50 [62.75–78.28]	0.003
Cognitive functioning (CF)	100.00 [89.12–55.31]	100.00 [88.47–96.14]	0.16
Social functioning (SF)	50.00 [29.48–40.39]	50.00 [37.03–46.31]	<0.001
Symptom scales			
Fatigue (FA)	22.22 [24.73–35.09]	22.22 [24.22–34.33]	0.18
Nausea and vomiting (NV)	0.00 [0.43–3.42]	0.00 [0.43–3.42]	1.0
Pain (PA)	16.67 [24.47–31.94]	16.67 [24.02–31.75]	0.53
Dyspnoea (DY)	0.00 [5.93–14.58]	0.00 [5.55–14.58]	0.70
Insomnia (SL)	33.33 [42.74–58.54]	33.33 [42.74–58.54]	1.0
Appetite loss (AP)	0.00 [7.08–15.99]	0.00 [7.08–15.99]	1.0
Constipation (CO)	0.00 [12.51–25.94]	0.00 [11.21–24.69]	0.16
Diarrhoea (DI)	0.00 [5.37–13.86]	0.00 [6.50–15.29]	0.16
Financial difficulties (FI)	0.00 [5.93–14.58]	0.00 [4.81–13.13]	0.32
CXC-24			
Functional scale			
Body image (BI)	33.33 [20.25–31.04]	33.33 [23.14–33.27]	0.01
Sexual activity (SXA)	33.33 [12.63–21.99]	33.33 [24.72–36.82]	<0.001
Sexual enjoyment (SXE)	0.00 [8.26–17.38]	16.67 [14.52–27.78]	0.01
Sexual vaginal functioning (SXV)	41.67 [39.28–50.46]	66.67 [60.44–69.37]	<0.001
Symptom scale			
Symptom experience (SE)	9.09 [10.71–14.93]	9.09 [9.39–12.86]	0.001
Lymphoedema (LY)	33.33 [26.34–33.92]	33.33 [27.16–34.37]	0.56
Peripheral neuropathy (PN)	0.00 [5.79–16.00]	0.00 [4.43–13.51]	0.10
Menopausal symptoms (H)	66.67 [43.79–62.62]	66.67 [45.67–63.31]	0.92
Sexual worry (SW)	66.67 [68.96–79.75]	66.67 [46.73–63.52]	<0.001

Data are shown as median [95%, CI].
